# Mesoporous-silica-coated palladium-nanocubes as recyclable nanocatalyst in C–C-coupling reaction – a green approach†

**DOI:** 10.1039/d0ra02281k

**Published:** 2020-07-14

**Authors:** Darius Rohleder, Philipp Vana

**Affiliations:** Institute of Physical Chemistry, Georg-August-University Goettingen Tammannstr. 6 37077 Goettingen Germany pvana@uni-goettingen.de; Woehler Research Institute for Sustainable Chemistry (WISCh), Georg-August-University Goettingen Tammannstr. 2 37077 Goettingen Germany

## Abstract

We report the straightforward design of a recyclable palladium-core–silica-shell nanocatalyst showing an excellent balance between sufficient stability and permeability. The overall process – design, catalysis and purification – is characterized by its sustainability and simplicity accompanied by a great recycling potential and ultra high yields in C–C-coupling reactions.

## Abstract 2

A green approach: in a single-step coating process a mesoporous silica shell was tailored onto palladium-nanocubes. Along with a PEG-matrix this core–shell-nanocatalyst could be recovered after C–C-coupling reactions and reused without any significant decrease in product yield.

## Graphical abstract

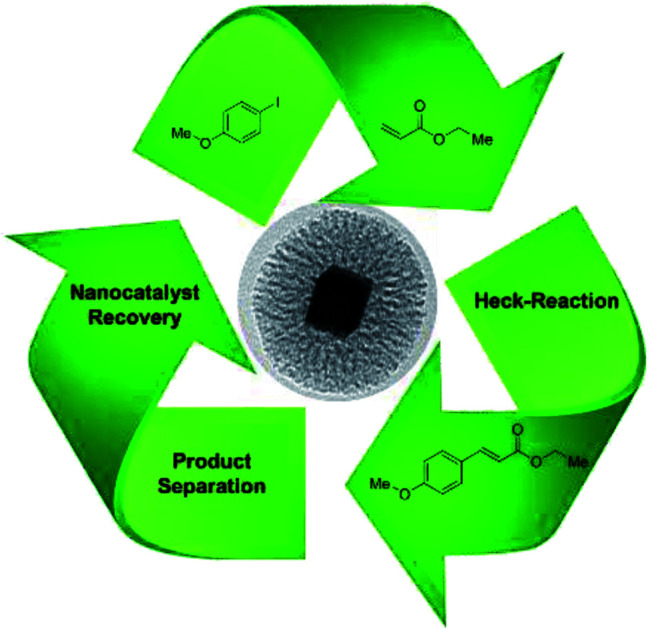

In contrast to bulk materials, metal-nanocyrstals (NC) possess unique physical and chemical properties. Both, nano-scale and geometry can dictate their optical, electronic and catalytic behavior.^1–3^ Consequently, nanomaterials have been implemented already in various fields like medicine,^4^ sensing,^5^ nano-electronics^6^ and organic synthesis.^7^ Smart strategies for a sustainable usage of limited resources such as precious metals and hydrocarbons are inevitable due to the consistently growing population and economy.^8–10^ Generally, catalysis gives rise to novel and energy-saving synthetic routes. However, homogeneous catalysis is not widely used in industrial processes, due to the need of mostly toxic ligands and the costly purification along with a restricted reusability potential.^11–13^ Heterogeneous catalysis based on the utilization of metal nanocrystals overcomes most of these limitations. Caused by its high surface-to-volume ratio, catalytic activities are drastically increased in comparison to bulk materials. However, the most common disadvantage in NC-based catalysis lies in the occurrence of aggregates during the reaction, which leads to a decrease of the catalytic active surface. Several studies were already presented in literature to delay that phenomenon using micelle-like- or core–shell-nanostructures as potential nanocatalytic systems.^14–20^ However, these surfactants or shells are either potentially harmful for the environment or very step-inefficient to produce. Other approaches used the deposition of small NCs in a mesoporous support which offers a large active surface area.^21–24^ In this context, it is crucial to find an adequate balance between permeability for small organic molecules and the overall stability of the nanocatalyst. Overcoming these obstacles can contribute to a sustainable supply of drugs and other organic substances.

In the present work palladium-nanocubes (Pd-NCubes) were fabricated in aqueous solution using cetyltrimethyl-ammoniumbromide (CTAB) as surfactant. The procedure is adapted from a previously published study.^25,26^ The respective transmission electron microscopy (TEM) image and the selected area electron diffraction (SAED) pattern of the as-obtained Pd-NCubes are depicted in Fig. S1.† The SAED confirms the single-crystallinity of Pd-NCubes bound by {100}-facets. TE micrographs of Pd-NCubes revealed an average edge-length of (18 ± 2) nm (for histogram see Fig. S2†). The formation of polyhedra and nanorods was found to be less than 1%.

To the best of our knowledge, there is no procedure reported that showed the direct fabrication of a mesoporous silica (mSi) shell tailored on Pd-NCs. However, Matsuura *et al.* demonstrated a single-step coating approach of CTAB-capped gold-nanorods and CdSe/ZnS quantum dots obtaining a mesoporous silica shell.^27^ The pores were determined to be 4 nm in width with 2 nm thick walls. Since the Pd-NCubes are covered by CTAB, already no surfactant exchange is necessary. Consequently, this procedure could be directly transferred to the as-obtained Pd-NCubes (∼10^15^ particles per L) of this study using tetraethyl orthosilicate (TEOS) as silica precursor in an alkaline solution. Here, CTAB serves as organic template for the formation of the mesoporous silica shell.

TE micrographs of individual Pd-mSi-nanohybrids are depicted in Fig. 1 showing a spherical silica coating with a thickness of (17 ± 2) nm (for histogram see Fig. S3†). The porosity is essential to ensure that vacant coordination sites on the palladium-core are present and accessible for catalysis. In contrast to other multistep approaches, pores are formed *in situ* with no additional etching step necessary.^28,29^ This avoids the usage of harmful etching agents such as fluorides or ammonia.^30^ The silica shell then served as platform for further surface modification using two different PEG-silanes (*M*_n_ = 5000 g mol^−1^ and *M*_n_ = 20 000 g mol^−1^). TE microscopy did not reveal any changes in the structure of the PEG functionalized Pd-mSi-nanohybrids opposed to the unfunctionalized nanocatalyst, since the contrast of polymer is too low (see Fig. S4†). However, dynamic light scattering (DLS) measurements in diluted aqueous solutions proved an increased hydrodynamic radius with increasing molecular weight of the PEG-chain grafted onto the silica shell (see Fig. 2). These results provide evidence that only individual nanostructures are formed while no larger aggregates are present.

**Fig. 1:**
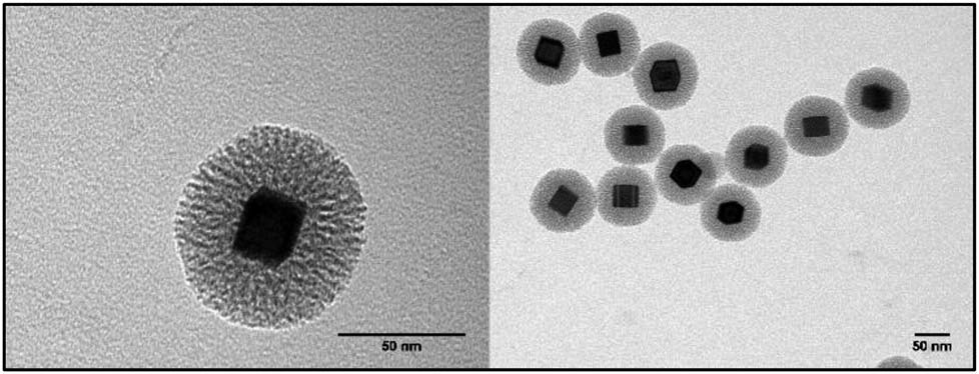
Exemplary TE micrographs of Pd-mSi-nanohybrids.

**Fig. 2:**
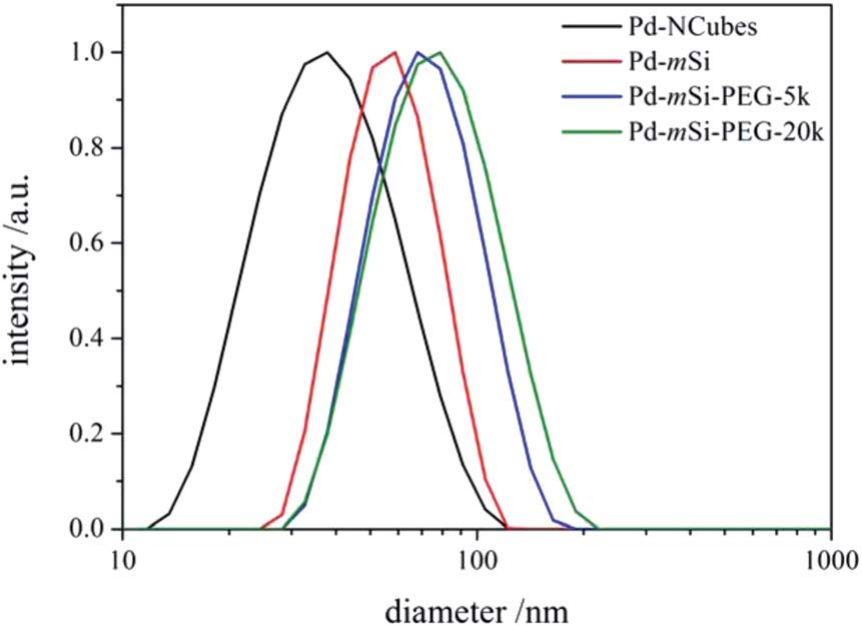
DLS results along the different stages of the hierarchal fabrication process of the nanocatalyst.

The successful functionalization of the Pd-mSi-nanohybrid with PEG provides the dispersibility for the overall nanocatalyst in a PEG matrix. Due to its lack of toxicity and its simple recovery, caused by its melting point at ∼50 °C, PEG is considered as a “green” reaction medium.^31^ It has already been shown that PEG can act as suitable solvent for both homogeneous and heterogeneous catalysis.^32–34^ Consequently, further experiments were performed using only the Pd-mSi-nanohybrid functionalized with PEG-silane having an average molecular weight of *M*_n_ = 5000 g mol^−1^ (PEG-5k). For catalytic reactions, Pd-mSi-PEG-5k was dispersed in a PEG matrix (PEG-2000, *M*_n_ = 2000 g mol^−1^) and charged into a Teflon centrifuge tube. Using only one tube for the reaction and the product separation avoids an additional transfer step and prevents any loss of the nanocatalyst between reaction cycles (see recycling Scheme 1). Here, the C–C-coupling between ethyl acrylate and *p*-iodoanisole served as model Heck-reaction to prove the catalytic activity of the designed catalyst (4.4 mol% overall Pd conc. equal to *ca.* 0.3 mol% surface-available Pd; conc. is determined by ICP-MS measurements, see Table S1†). Sodium phosphate was used as base providing the largest product yield when compared to other bases, such as Na_2_CO_3_, K_2_CO_3_ and K_3_PO_4_. Once the catalysis was performed and the PEG-2000 was cooled down, diethyl ether was added to extract the product and separated from the reaction medium *via* centrifugation.

**Scheme 1:**
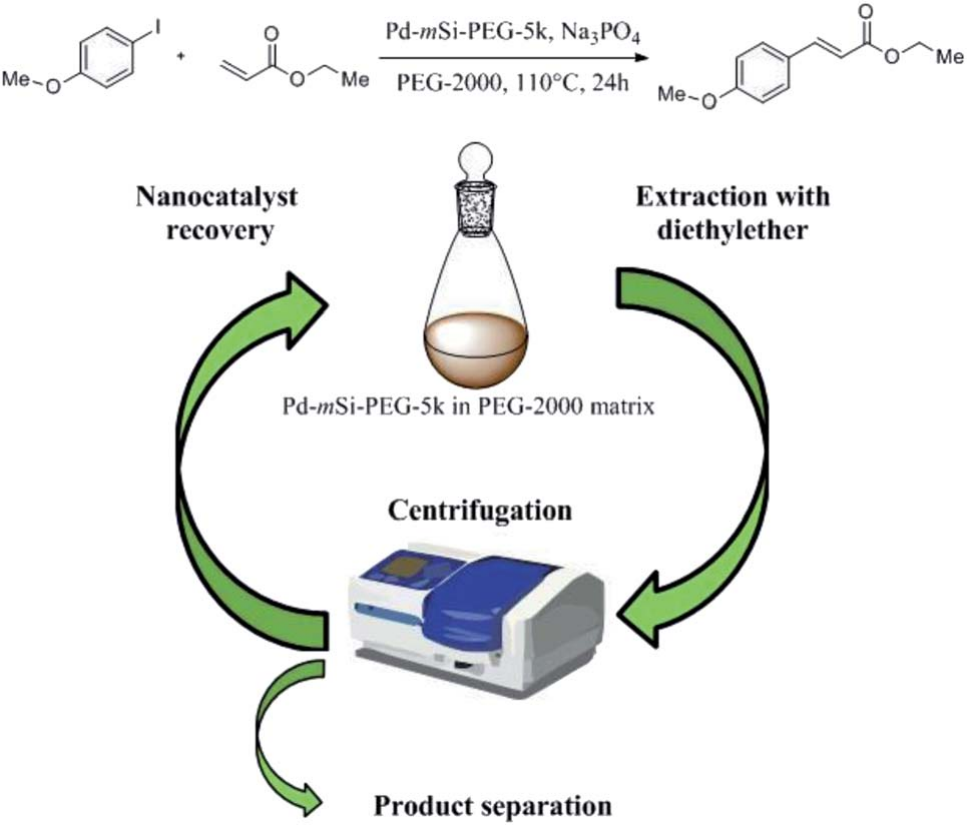
Recycling process of the Pd-mSi-PEG-5k-nanocatalyst and PEG-2000 as solvent after the Heck-reaction between ethyl acrylate and *p*-iodoanisole to form ethyl *p*-methoxycinnamate.

To exclude any suspended compounds from the desired product, the mixture was passed through a PTFE-filter. Et_2_O was removed *in vacuo* without the need of column chromatography. Comparing the ^1^H-NMR spectra of the as-obtained product with the educts indicate a yield of 98% with only small amounts of PEG-2000 present (identified by the signal at ∼3.6 ppm, < 1 weight-%). The results show that both educts and the base Na_3_PO_4_ are able to diffuse through the mesoporous silica shell to the palladium core (see Fig. 3). A detailed ^1^H-NMR signal assignment of the product is given in Fig. S5.†

**Fig. 3:**
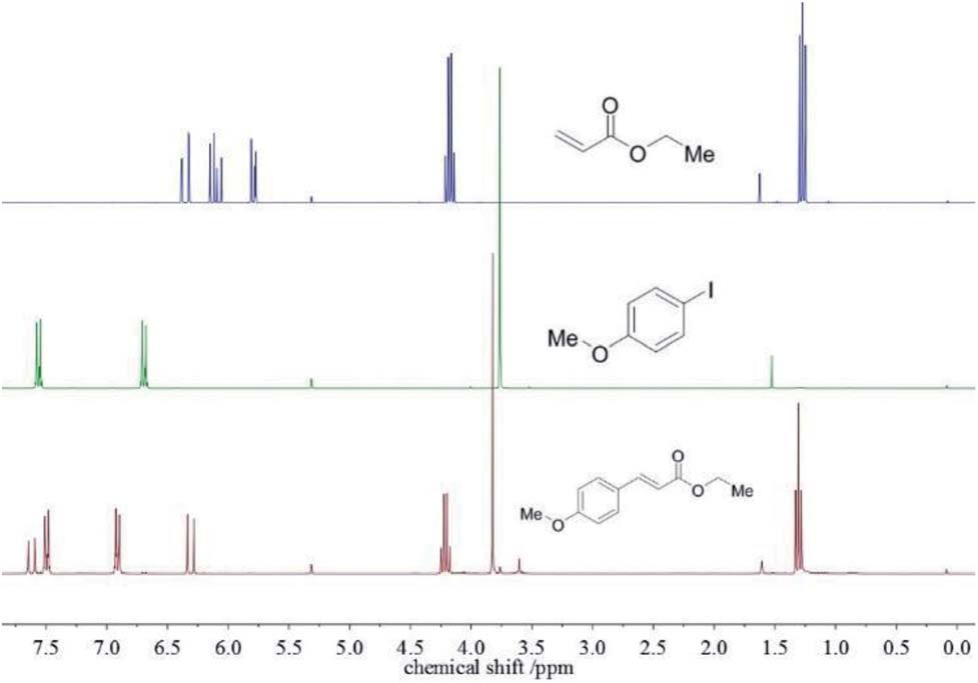
^1^H-NMR spectra of the educts ethyl acrylate (top) and *p*-iodoanisole (center) and the product ethyl *p*-methoxycinnamate (bottom).

After recovering the reaction mixture containing Pd-mSi-PEG-5k and PEG-2000, seven further Heck-reaction-cycles were conducted under the same conditions. Results obtained from ^1^H-NMR and gravimetry indicate no significant decrease in catalytic activity (see ^1^H-NMR spectra in Fig. 4). Along the eight Heck-reactions, product yields were determined between 94% and 99%. Only small traces of *p*-iodoanisole (identified by the signal at ∼6.75 ppm) were still present while ethyl acrylate could be fully removed *in vacuo*. The yields obtained after each cycle are displayed in Fig. 5. ICP-MS measurements of the catalysis product were performed to determine the palladium leaching out of the catalytic system. The results are displayed in Table S1† indicating that leaching is strongly suppressed since the overall Pd-content in the product ranges from 0.3–5.7 ng, only. This corresponds to 0.002–0.044 ppm palladium with respect to the product mass. The data are in good agreement with the high product yields along the eight Heck-reactions. To trace the evolution of the nanocatalyst along the cycles, TE micrographs were taken after the 1^st^ and the 8^th^ Heck-cycle (see Fig. 6). It can be seen that the cubical structure of the palladium vanishes during the first reaction (left TEM image). Inside the silica shell spherical palladium particles were formed. An explanation for this rearrangement lies in the suggested Heck-mechanism.^35^ Here, a Pd^2+^-species forms after the oxidative addition of the *p*-iodoanisole which can desorb from the Pd-NCube. Once the reductive elimination of the product occurs the Pd^0^-species is generated again that can re-deposit on the palladium-core.^21^ Since the spherical geometry possesses the lowest free surface energy, globules were eventually formed.^3^ The mesoporous silica shell is not affected significantly by the catalysis and the rearrangement of the palladium. The porosity appears to stay intact, enabling the penetration of further small organic molecules. Control experiments were performed to validate whether these observations are based on either a thermally or a chemically induced rearrangement process of the palladium core. Therefore, only the nanocatalyst was dispersed in PEG-2000 and heated at 110 °C for 24 h without any conducted catalysis reaction. TEM results showed no changes in the structure of neither the palladium core nor the silica shell (see Fig. S6†). After the 8^th^ reaction, no core–shell-nanostructures could be detected anymore *via* TEM. Only small randomly shaped Pd-nanoparticles (≤20 nm) were found indicating a slow leaching of the palladium out of the silica shell. However, no larger aggregates were formed (see right TE micrograph in Fig. 6) which explains the continuously high catalytic activity.

**Fig. 4:**
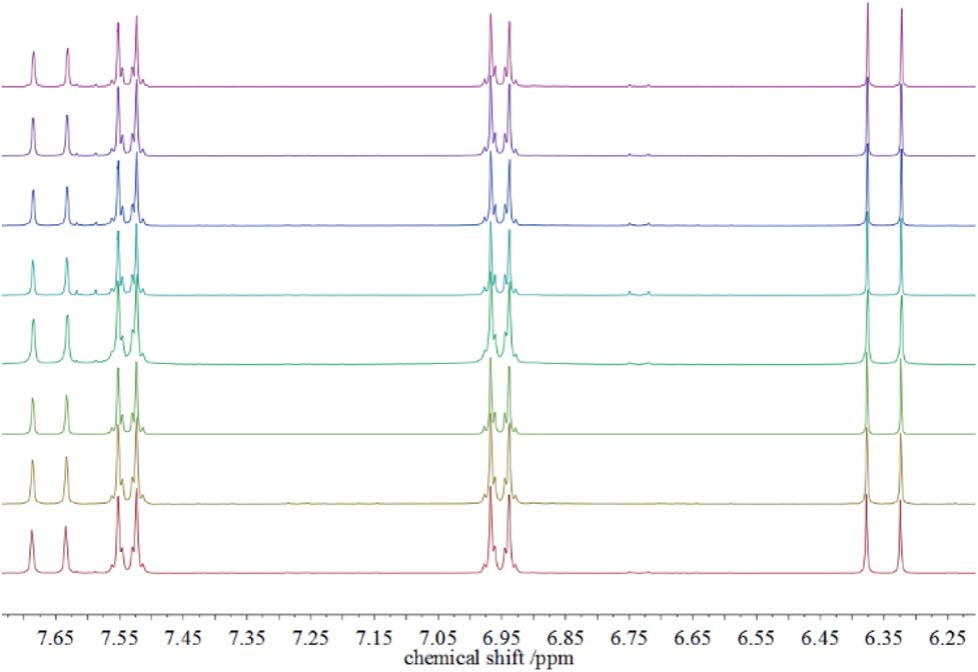
^1^H-NMR spectra of the product ethyl *p*-methoxycinnamate after the 1^st^ Heck-reaction (bottom) up to the 8^th^ reaction (top).

**Fig. 5:**
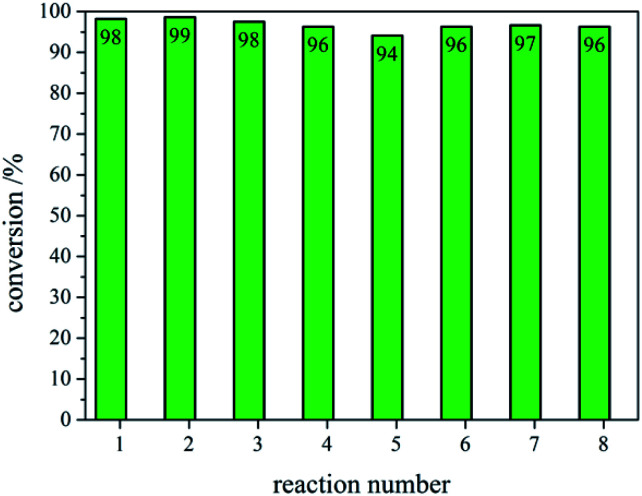
Conversions of ethyl *p*-methoxycinnamate obtained *via* NMR and gravimetry after each respective Heck-reaction (1–8).

**Fig. 6:**
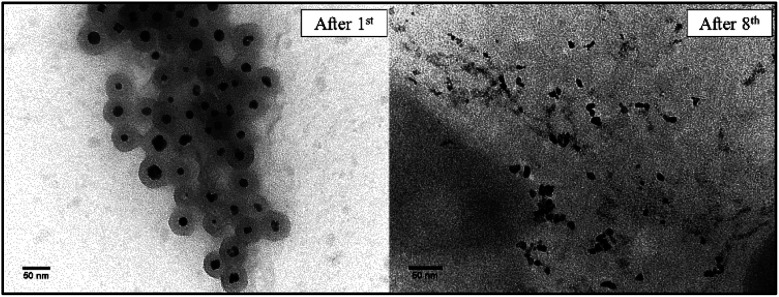
TE micrographs of the Pd-mSi-PEG-5k-nanocatalyst taken after the 1^st^ (left) and the 8^th^ Heck-reaction (right).

## Conclusions

In summary, we have presented an efficient design strategy to obtain a highly active, heterogeneous palladium nanocatalyst protected by a mesoporous silica shell. This core–shell-nanohybrid revealed an outstanding stability over at least several months in the presence of both water and air. During catalysis the mSi-shell showed sufficient permeability for organic molecules and thermal stability at elevated temperatures. It could be demonstrated that both the catalyst and the solvent can easily be recycled after Heck-reactions and subjected to further reaction-recovery-cycles without any significant decrease in catalytic activity. The newly developed approach offers various advantages over other catalytic systems: 1^st^ the highly step-efficient and simple fabrication process of both the Pd-catalyst and its shell, 2^nd^ the absence of expensive and toxic ligands and solvents, 3^rd^ the full recyclability of the nanocatalyst and the reaction medium and 4^th^ the facile isolation of the product without further need of chromatographic purification to receive excellent product purities. Consequently, this work may contribute substantially to a more sustainable usage of limited resources.

## Conflicts of interest

The authors declare no conflict of interest.

## Supplementary Material

RA-010-D0RA02281K-s001

## References

[cit1] Ghosh S. K., Pal T. (2007). Chem. Rev..

[cit2] Jain P. K., Eustis S., El-Sayed M. A. (2006). J. Phys. Chem. B.

[cit3] Narayanan R., El-Sayed M. A. (2005). J. Phys. Chem. B.

[cit4] Rai M., Ingle A. P., Birla S., Yadav A., Dos Santos C. A. (2016). Crit. Rev. Microbiol..

[cit5] Vilela D., González M. C., Escarpa A. (2012). Anal. Chim. Acta.

[cit6] Homberger M., Simon U. (2010). Philos. Trans. R. Soc., A.

[cit7] Astruc D. (2020). Chem. Rev..

[cit8] Ludwig J. R., Schindler C. S. (2017). Chem.

[cit9] Nakajima K., Daigo I., Nansai K., Matsubae K., Takayanagi W., Tomita M., Matsuno Y. (2018). Resour., Conserv. Recycl..

[cit10] Vidal O., Rostom F., François C., Giraud G. (2017). Elements.

[cit11] Lyons T. W., Sanford M. S. (2010). Chem. Rev..

[cit12] Yin L., Liebscher J. (2007). Chem. Rev..

[cit13] Ackermann L. (2011). Chem. Rev..

[cit14] Pontes da Costa A., Nunes D. R., Tharaud M., Oble J., Poli G., Rieger J. (2017). ChemCatChem.

[cit15] Papagiannouli I., Demetriou M., Krasia-Christoforou T., Couris S. (2014). RSC Adv..

[cit16] Shi S. P., Zhang L. F., Zhu J., Zhang W., Cheng Z. P., Zhu X. L. (2009). eXPRESS Polym. Lett..

[cit17] Reetz M. T., Westermann E. (2000). Angew. Chem., Int. Ed..

[cit18] Beletskaya I. P., Kashin A. N., Litvinov A. E., Tyurin V. S., Valetsky P. M., van Koten G. (2006). Organometallics.

[cit19] Ogasawara S., Kato S. (2010). J. Am. Chem. Soc..

[cit20] Keller M., Hameau A., Spataro G., Ladeira S., Caminade A. M., Majoral J. P., Ouali A. (2012). Green Chem..

[cit21] Biffis A., Centomo P., Del Zotto A., Zecca M. (2018). Chem. Rev..

[cit22] Kalbasi R. J., Negahdari M. (2014). J. Mol. Struct..

[cit23] Mehnert C. P., Weaver D. W., Ying J. Y. (1998). J. Am. Chem. Soc..

[cit24] Mehnert C. P., Ying J. Y. (1997). Chem. Commun..

[cit25] Niu W., Li Z. Y., Shi L., Liu X., Li H., Han S., Chen J., Xu G. (2008). Cryst. Growth Des..

[cit26] Niu W., Zhang L., Xu G. (2010). ACS Nano.

[cit27] Gorelikov I., Matsuura N. (2008). Nano Lett..

[cit28] Graf C., Vossen D. L. J., Imhof A., van Blaaderen A. (2003). Langmuir.

[cit29] Werner Stöber E. B., Fink A. (1968). J. Colloid Interface Sci..

[cit30] Chen Y., Chen H. R., Shi J. L. (2014). Acc. Chem. Res..

[cit31] Chen J., Spear S. K., Huddleston J. G., Rogers R. D. (2005). Green Chem..

[cit32] Luo C., Zhang Y., Wang Y. (2005). J. Mol. Catal. A: Chem..

[cit33] Ackermann L., Vicente R. (2009). Org. Lett..

[cit34] Vafaeezadeh M., Hashemi M. M. (2015). J. Mol. Liq..

[cit35] Heck R. F., Nolley J. P. (1972). J. Org. Chem..

