# Supplementary material for: Mesoporous-silica-coated palladium-nanocubes as recyclable nanocatalyst in C–C-coupling reaction – a green approach
Source: RSC Adv. 2020 Jul 14;10(44):26504–7. doi: 10.1039/d0ra02281k (PMC9055435; doi:10.1039/d0ra02281k)
Supplement: RA-010-D0RA02281K-s001 [file RA-010-D0RA02281K-s001.pdf]

Electronic Supplementary Material (ESI) for RSC Advances.
This journal is © The Royal Society of Chemistry 2020

Supplementary Information

Mesoporous-Silica-Coated Palladium-Nanocubes as Recyclable Nanocatalyst in C–C-Coupling Reaction – A Green Approach

Darius Rohleder and Philipp Vana

We report the straightforward design of a recyclable palladium-core–silica-shell nanocatalyst showing an excellent balance between sufficient stability and permeability. The overall process – design, catalysis and purification – is characterized by its sustainability and simplicity accompanied by a great recycling potential and ultra high yields in C–C-coupling reactions.

Table of Contents

- Experimental Procedures 3
    - Materials 3
    - Analytical methods 3
    - Fabrication of CTAB-capped Pd-NCubes 3
    - Mesoporous silica coating on Pd-NCubes 4
    - PEG-functionalization of Pd-*m*Si-nanohybrids and preparation of the nanocatalytic system 4
    - Heck-cycles using Pd-*m*Si-PEG-5k as nanocatalyst 4

- Results and Discussion 4
    - TEM image and SAED pattern of CTAB-capped Pd-NCubes 4
    - Histogram of Pd-Ncube edge-length distribution 5
    - Histogram of mesoporous silica shell-thickness distribution 5
    - Exemplary TE micrographs of Pd-*m*Si-PEG-5k nanohybrids 6
    - ^{1}H-NMR of ethyl *p*-methoxycinnemate 6
    - Determination of Pd-content within the nanocatalyst and the catalysis product 7
    - TEM images of thermal control experiments of Pd-*m*Si-PEG-5k 7

- References 7

Experimental Procedures

Materials

Ascorbic acid (99.5%), cetyltrimethyl ammonium chloride (CTAB, >99.0%), ethanol (>99.8%), diethyl ether (≥ 98%), (hydrochloric acid solution (1 M), methanol (>99.9%), (palladium chloride (99%), polyethylene glycol (PEG, M_n = 2,000 g mol^{-1}), PEG-silanes (M_n = 5,000 g mol^{-1} and M_n = 20,000 g mol^{-1}), sodium hydroxide solution (0.1 M), sodium phosphate (96%) and tetraethyl orthosilicate (TEOS, 99.999%) were purchased from Sigma Aldrich. *p*-Iodoanisole (>99.0%) was purchased from TCI. If not indicated otherwise all chemicals were used as received. Ethyl acrylate (99%, Sigma Aldrich) was passed through a short column of inhibitor remover before used in catalytic reactions.
Nanopure water (type I) was obtained from a Millipore water purification system equipped with a UV lamp at an electrical resistivity of 18.2 MΩ cm and filtered through a 0.05 μm cellulose filter before used.

Analytical methods

Transmission electron microscopy (TEM) experiments were performed on a Philips CM 12 TEM operating at an acceleration voltage of 120 kV and an emission current of 3-4 μA. The focused electron spot has a diameter of 1 μm. Within the condenser lens a 50 μm aperture was used. To block all scattered electrons a aperture with a diameter of 20 μm was employed. An Olympus 1376 x 1032 pixel CCD camera served as detector. All samples were prepared by drop-casting and slow evaporation of the solvent. Samples were given on a Plano 200 mesh copper TEM grid holding an amorphous carbon film. These grids were placed on a paper tissue and cast by one drop using a Eppendorf-pipette. The grid was immediately covered by a glass vial to ensure slow evaporation. TE micrographs were analyzed by the software ImageJ.

Dynamic light scattering (DLS) measurements were conducted on a Malvern Zetasizer Nano S system using a wavelength of 633 nm at a scattering angle of 173°. Diluted aqueous solutions of the samples were prepared 24 h prior measurements and left in the dark without moving. Samples were measured 3 times for 60 s each at 25°C after a 120 s equilibration period within the analyzer.

Nuclear magnetic resonance (NMR) spectra were taken on a Bruker AMX-300. Samples were prepared in deuterated dichloromethane (~20 mg mL^{-1}). The spectra were referenced to the residual signal of the solvent.

Inductively coupled plasma – mass spectrometry (ICP-MS) measurements were performed on a ThermoFisher Scientific iCAP-Q ICPMS equipped with a Peltier-cooled cyclonic quartz glass spray chamber and a PFA microconcentric nebulizer operating at a flow rate of 100 μL min^{-1}. Solutions to by analyzed were supplied with an Elemental Scientific SC-FAST system which was connected to an Elemental Scientific SC-DX autosampler. Palladium-specific calibration measurements were conducted to guarantee an adequate data quality.
Heck-reaction samples were prepared by solving the catalysis product in diethyl ether (5 mL) and extracting potential residues of palladium with 0.5 M HCl (5 x 2 mL).

Fabrication of CTAB-capped Pd-NCubes

Prior any nanocrystal syntheses all glassware including the stirring bar and stoppers were cleaned using *aqua regia* and rinsed by demineralized water, acetone and nanopure water, respectively. Then the equipment was oven-dried at 130°C over night.

According to *Xu et al.* Pd-NCubes can be fabricated using CTAB) as surfactant and ascorbic acid as reducing agent.[1,2] Attempts to scale up the reaction were successful using the 10-fold amount of each chemical opposed to the reported process. In a typical synthesis, CTAB (455 mg, 125 mmol, 2500 eq.) was solved in nanopure water (95 mL) and heated at 95°C for 5 min. Under vigorous stirring, 5 mL of a 10 mM H_2PdCl_4 solution (12.5 mg, 0.05 mmol, 1.0 eq.) was added. Meanwhile, a freshly prepared ascorbic acid solution (100 mM) was also heated at 95°C. After 5 min 800 μL of the ascorbic acid solution (14.1 mg, 0.08 mmol, 1.6 eq.) was given to the metal solution. Within 10 s the solution turned from orange to brown. The reaction was allowed to proceed for further 30 min at a slower stirring rate. The as-obtained solution was stored in PP tubes in the dark at room temperature. TE micrographs of the Pd-NCubes revealed an edge-length of (18 ± 2) nm (for histogram see Fig. S2). Opposed to the work of *Xu et al.* the formation of polyhedra and nanorods was reduced to less than 1%.

Mesoporous silica coating on Pd-NCubes

Matsuura and *Gorelikov* outlined a general procedure to coat CTAB-capped nanoparticles with a mesoporous silica shell.[3] Herein, a single-step synthesis procedure was performed using Au-NRs and CdSe/ZnS quantum dots. This approach was transferred to CTAB-capped Pd-NCubes. To remove the excess of surfactant (CTAB) from the as-obtained Pd-NCubes 20-25 mL of the Pd-sol were centrifuged at 15,000 g at 30°C for 30 min. After discarding the supernatant the precipitate was redispersed in nanopure water (10 mL) to yield a concentration of ~10^{15} particles per liter. Under smooth shaking a 0.1 M solution of NaOH (100 μL) was added. Afterwards, two injections of pure TEOS (2 x 6 μL) were introduced at 30 min time intervals under gentle vortexing. Then, the mixture was incubated for 2 days without moving at room temperature. TEM images of the Pd-*m*Si-nanohybrids showed a shell thickness of (17 ± 2) nm (for histogram see Fig. S3).

PEG-functionalization of Pd-*m*Si-nanohybrids and preparation of the nanocatalytic system

The respective PEG-silane (M_n = 5,000 g mol^{-1} and M_n = 20,000 g mol^{-1}) was solved in a mixture of ethanol and nanopure water (10:1, 10 mg mL^{-1}). Then, 1 mL of this solution was given directly to the Pd-*m*Si-nanohybrid solution under ultrasonication. The mixture was incubated over night under vigorous shaking. Afterwards, two aliquots were combined to yield ~2.6 mg palladium per sample (determined by ICP-MS). To remove the solvent the mixture was centrifuged at 12,000 *g* (30°C) for 60 min and the supernatant was discarded. The precipitate was redispersed in methanol under ultrasonication. PEG-2000 (1 g) was solved in this solution and the whole mixture was precipitated into a Teflon tube filled with an excess of cold diethyl ether. This suspension was centrifuged for 20 min (5°C) at 6,000 *g* and the supernatant was discarded afterwards. To remove residuals of diethyl ether the Teflon tube was degassed and then transferred to glove box (argon).

Heck-cycles using Pd-*m*Si-PEG-5k as nanocatalyst

A typical synthesis was conducted as follows: To the Pd-*m*Si-PEG-5k (~2.6 mg palladium, 0.024 mmol, 0.044 eq.) dispersed in PEG-2000 (1.0 g, 1.0 mol, 1800 eq.) *p*-iodoanisole (130 mg, 0.56 mmol, 1.0 eq.), ethyl acrylate (110 mg, 1.1 mmol, 2.0 eq.) and sodium phosphate (180 mg, 1.1 mmol, 2.0 eq.) was given. The Teflon tube was then transferred to an aluminum bead bath and heated at 110°C for 24 h. The mixture was vigorously stirred over the period of the reaction. To shut down the reaction the tube was removed from the bath and cooled down. Afterwards, diethyl ether (20 mL) was added to the mixture and stirred for 15 min to extract the product. This suspension was centrifuged at 10°C for 20 min at 6,000 *g*. The supernatant was removed by a syringe and passed through a PTFE filter (poresize: 0.45 μm) before the diethyl ether and the residual ethyl acrylate was removed *in vacuo*. This extraction process was conducted two further times. The Pd-*m*Si-PEG-5k nanocatalyst and the PEG-2000 was recovered and degassed afterwards. Finally, the mixture was transferred to the glove box again to conduct the next Heck-reaction. Likewise, seven further catalysis–recovery-cycles were performed.

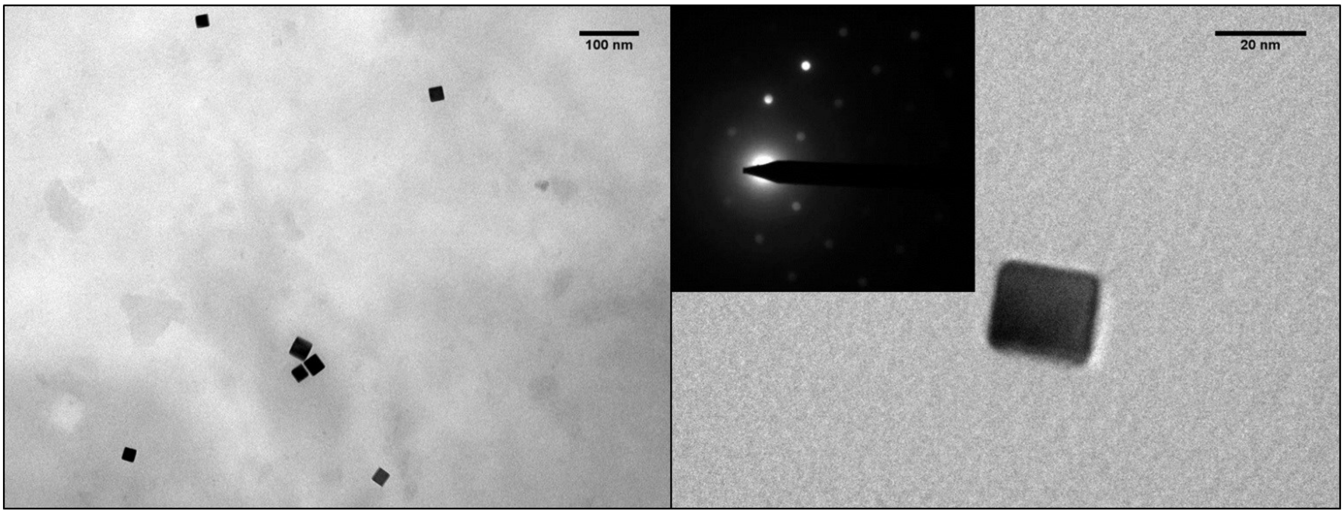

Fig. S1. Exemplary TE micrographs and selected area electron diffraction (SAED) pattern of CTAB-capped Pd-NCubes obtained by an upscaled fabrication process adapted from Xu et al.

Results and Discussion

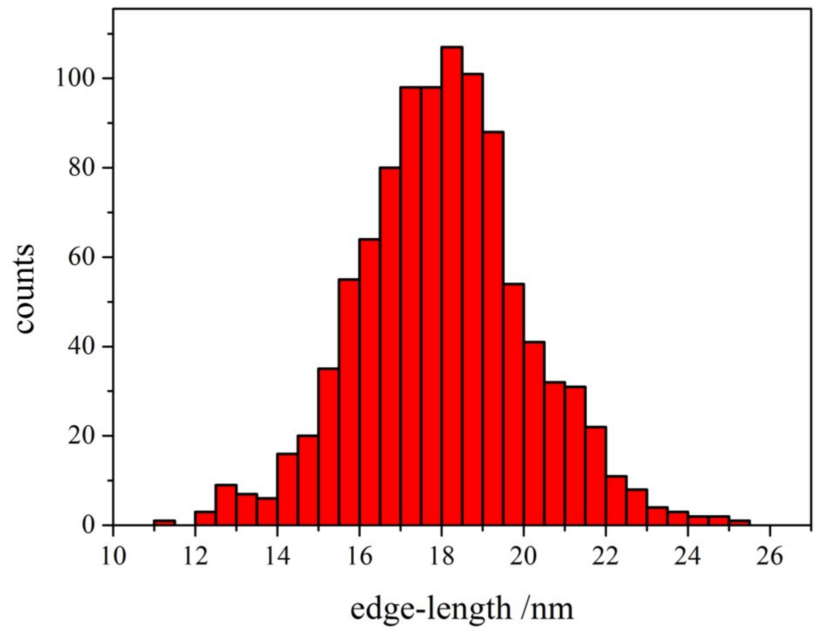

Fig. S2. Size distribution of Pd-NCube edge-lengths obtained from multiple TE micrographs.

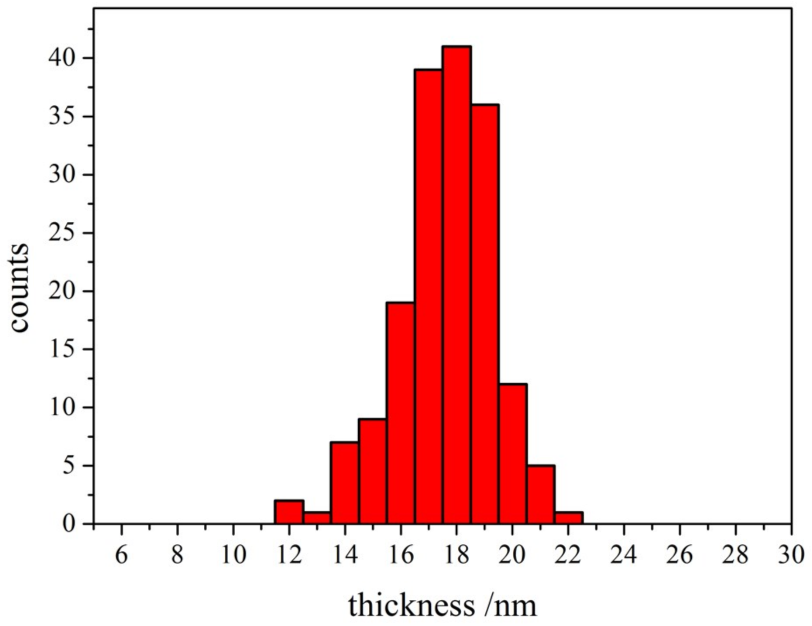

Fig. S3. Size distribution of the mesoporous silica shell-thickness coated on CTAB-capped Pd-NCubes.

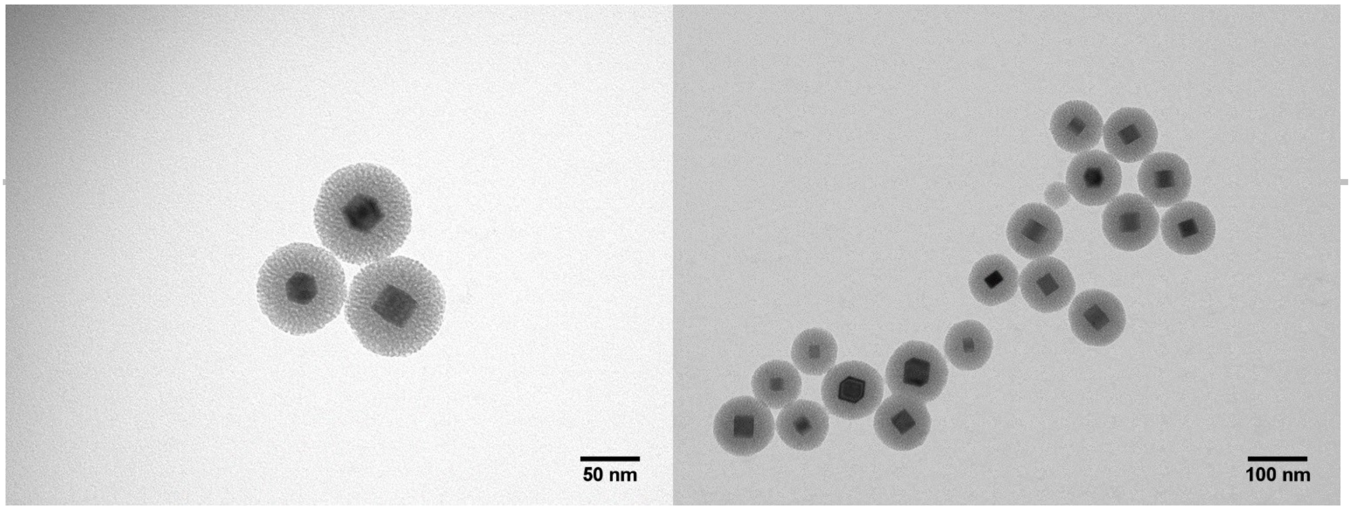

Fig. S4. Exemplary TE micrographs of Pd-*m*Si-PEG-5k nanohybrids.

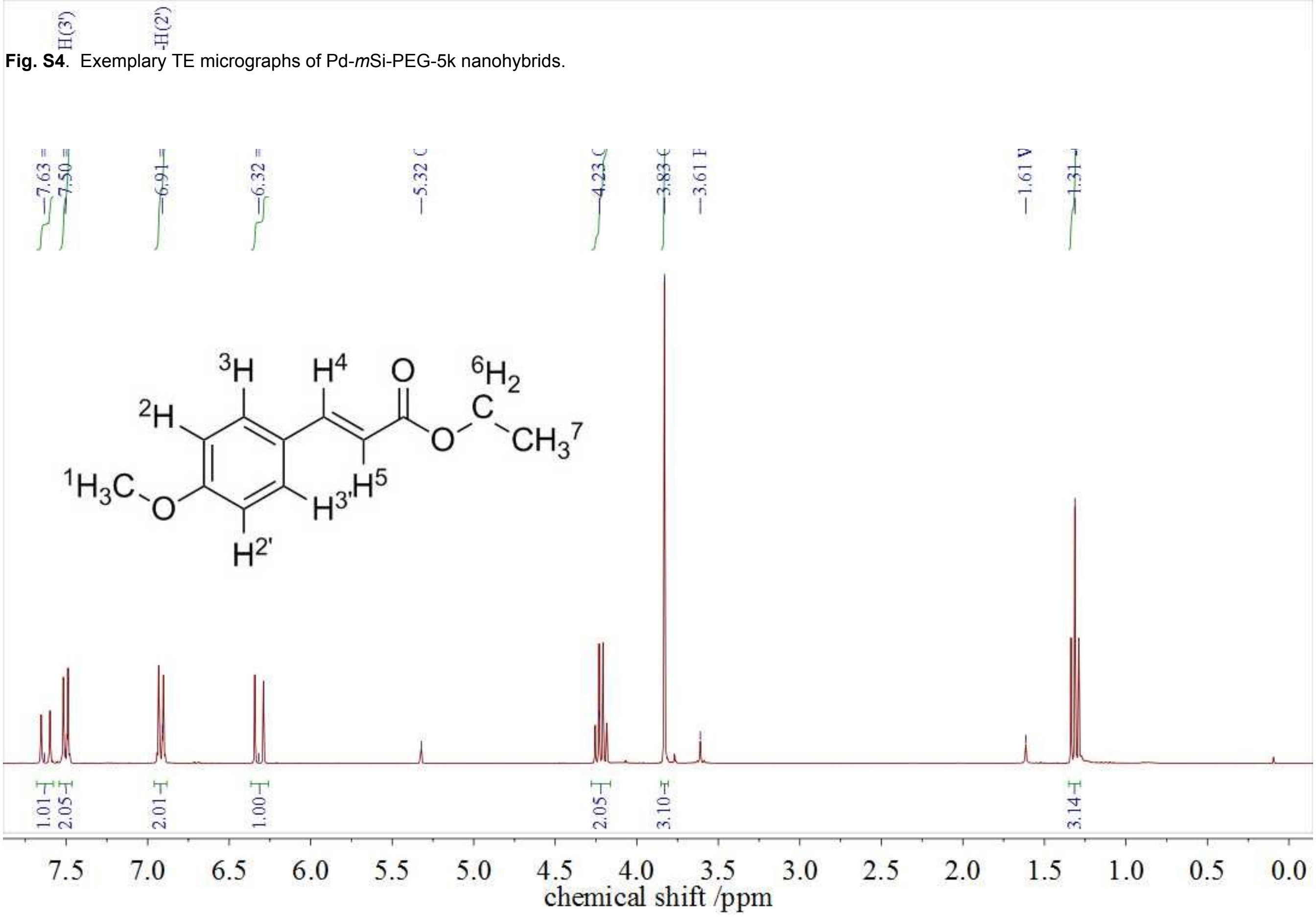

Fig. S5. ^{1}H-NMR spectrum of ethyl p-methoxycinnemate obtained from the palladium-catalyzed Heck-reaction and the assignment of the respective signals to the chemical structure.

Tab. S1. Results of ICP-MS measurements to determine the palladium content within the nanocatalyst (Pd-*m*Si-PEG-5k) and within the extracted catalysis product. The values in brackets corresponds to the amount of palladium with respect to the overall product mass.

**Sample**	**Pd-content**
**Pd-*m*Si-PEG-5k (nanocatalyst)**	2.6 mg
**1. Heck-reaction**	5.1 ng (0.044 ppm)
**2. Heck-reaction**	5.0 ng (0.038 ppm)
**3. Heck-reaction**	4.2 ng (0.032 ppm)
**4. Heck-reaction**	1.5 ng (0.012 ppm)
**5. Heck-reaction**	5.7 ng (0.044 ppm)

**6. Heck-reaction**	1.7 ng (0.013 ppm)
**7. Heck-reaction**	1.6 ng (0.014 ppm)
**8. Heck-reaction**	0.3 ng (0.002 ppm)

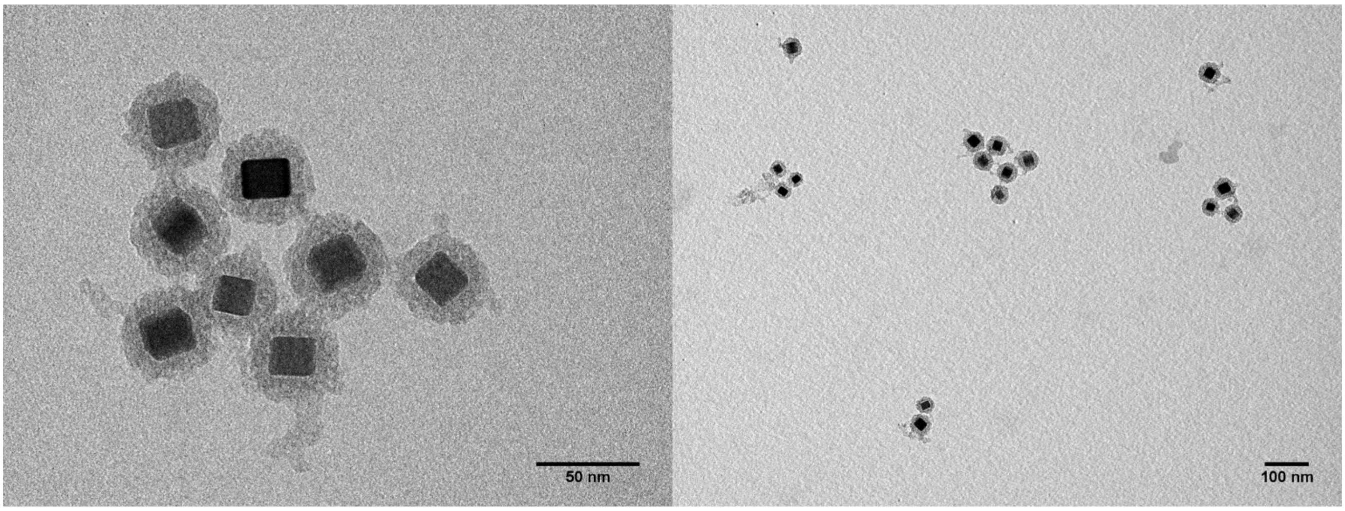

Fig. S6. TE micrographs of the Pd-mSi-PEG-5k taken after heating the nanocatalyst in PEG-2000 under Argon at 110°C for 24 h without performing any Heck-reaction.

References

1 W. Niu, Z. Y. Li, L. Shi, X. Liu, H. Li, S. Han, J. Chen and G. Xu, *Cryst. Growth Des.*, 2008, **8**, 4440–4444.
2 W. Niu, L. Zhang and G. Xu, *ACS Nano*, 2010, **4**, 1987–1996.
3 I. Gorelikov and N. Matsuura, *Nano Lett.*, 2008, **8**, 369–373.
